# A Vivisection Bill

**Published:** 1908-02

**Authors:** 


### A Vivisection Bill.

A YOUNG man with a pleasing presence is making the rounds of physicians' offices seeking support for a bill for the supervision of vivisection in this State. It is apparent from an examination of the bill that the effort to regulate so-called vivisection is made in honesty and good faith; but, like many other reform measures it is an unnecessary bit of trimming, for it purposes to put an end to a state of affairs which does not obtain, that is, the inflicting of pain on animals in scientific investigation.

The horrible and soul-harrowing pictures painted by antivivisection cranks and antivaccinationists are purely products of overwrought imaginations in the great majority of cases; and, while the bill under consideration, even if passed in its present form. would have little or no effect on the progress of scientific experimentation, it has vicious shadows and is merely the opening wedge for more arbitrary and fool legislation.

It is suggested that before signing the petition for the passage of the measure physicians give it a careful reading and a more careful consideration in all its phases.

AMBASSADOR REID'S address, published elsewhere, delivered during the midwinter meeting of the New York State Teachers' Association at Syracuse, deserves attentive reading by every parent, educator, teacher, or public-spirited citizen. Especially would we invite the attention of teachers to what Mr. Reed says under the heading, "Suggestive Facts for Americans." There is no more disquieting location than the environment of a public school in Buffalo, particularly after the pupils have been dismissed. Lawlessness, heedlessness and all forms of unearthly noises run riot at such times. It is not only disturbing to the sick who may chance to reside in the neighborhood, but it is also almost equally distracting to well persons. We commend the subject to Mr. Superintendent Emerson, and to his subordinate principals, as well worthy their attention.

---

THE operation of appendicitis has been performed under many trying circumstances by surgeons of experience; but we read of such an operation lately performed at sea which overshadows all others regarding perplexing environment. It appears that the Cunard liner Pannonia, which left Gibraltar for New York on December 16, 1907, was the scene of this most trying ordeal. One of the stokers of the vessel was suddenly seized with severe pain a few days after the steamer left port and after being temporised with by the usual methods of the stoke room and the engineer's department, his condition was brought to the notice of the ship surgeon. After a somewhat hasty examination, Dr. J. Francis Orr diagnosticated appendicitis and immediately prepared the patient for operation. This was made while the ship was rolling and pitching under the influence of a heavy sea and, although braced and strapped to the operating table, the patient's body slid back and forth rendering it almost impossible for Dr. Orr to make an incision. He sent a request to the captain to bring the Pannonia to a dead stop until he could perform the operation. The strokes of the knife were made between lurches and within 35 minutes the operation was completed. When the ship reached New York, the stoker was convalescent.

---

A MEETING in reference to tuberculosis, under the auspices of the State Charities Aid Association and the State Commissioner of Health, was held at Harmanus Bleecker Hall, Albany, Monday evening, January 27, 1908, at 8.30. This meeting was held in connection with the one hundred and second annual meeting of the Medical Society of the State of New York and was presided over by the Hon. Joseph H. Choate. Addresses were made by Governor Charles E. Hughes, Lieutenant Governor Lewis Stuyvesant Chanler, Dr. William H. Welch, Dr. E. H. Porter, State Commissioner of Health, Hon. James W. Wadsworth, Jr., Senator W. W. Armstrong, Hon. Homer Folks, and others. This meeting formed part of a campaign being waged for the enlightenment of the public in relation to the prevention of tuberculosis. It should serve to unite public spirited citizens, physicians and philanthropic men and women in large numbers to act in concert upon this all important question.

---

THE decision of the navy department that hospital ships would be placed under the control and command of medical officers of the navy, while their navigation shall be exclusively controlled by a competent sailing-master and civilian crew, leaves small occasion for criticism or discussion. There is no reason for navy officers of the line to find fault with the plan because they are not subordinated to officers of the medical staff by this decision.

In view of this action a telegram to the Surgeon-General of the navy that his action meets the approval of 140,000 physicians of the United States is equally out of place. A medical officer has always been in charge of sick and wounded whether upon land or sea. The only point worthy of consideration is in relation to the actual direction of the movements of a hospital ship while in commission. Under the new rule orders will be transmitted direct to the medical officer in command of the "Relief" and he in turn directs the sailing-master, a civilian, what to do. And that is all there is of it.

In relation to this subject the *Washington Post* observes: that "the point that the navy's colliers ought to be commanded by coal dealers also seems to be well taken, but not many of them might care to exchange the rank of coal baron for that of rear-admiral."

---

A DEPARTMENT of Health to be presided over by a cabinet officer has been agitated as a question of propriety by the medical profession, or at least a portion of it, for some time past. The JOURNAL has been of the opinion that it would not be feasible to create such an office at present. One of the strong reasons for this opinion is the fact that the country is already somewhat over-burdened with officials, departments and bureaus; and especially is the present cabinet quite large enough for all practical purposes; to increase it in numbers is liable to make it unwieldy.

President Rossevelt recently declared his opinion in the premises, according to press dispatches, as follows:

I believe that we could with advantage have a bureau of health to be put under one of the existing departments, but we need no additional cabinet officers. On the contrary, they would be a disadvantage. While we do most urgently need a rearrangement of the bureaus and divisions of the present cabinet, we also need to have every executive office of the government put under some cabinet officer. I am against the creation of any independent bureau not under a cabinet office.

It may prove wise, in the near future, for Congress to create the office of under secretary for some of the departments; in which case an under secretary of the interior might assume charge of the public health.

